# High therapeutic efficacy of Cathelicidin-WA against postweaning diarrhea via inhibiting inflammation and enhancing epithelial barrier in the intestine

**DOI:** 10.1038/srep25679

**Published:** 2016-05-16

**Authors:** Hongbo Yi, Lin Zhang, Zhenshun Gan, Haitao Xiong, Caihua Yu, Huahua Du, Yizhen Wang

**Affiliations:** 1 Institute of Feed Science, College of Animal Science, Zhejiang University, 866 Yuhangtang Road, Hangzhou, Zhejiang 310058, China

## Abstract

Diarrhea is a leading cause of death among young mammals, especially during weaning. Here, we investigated the effects of Cathelicidin-WA (CWA) on diarrhea, intestinal morphology, inflammatory responses, epithelial barrier and microbiota in the intestine of young mammals during weaning. Piglets with clinical diarrhea were selected and treated with saline (control), CWA or enrofloxacin (Enro) for 4 days. Both CWA and Enro effectively attenuated diarrhea. Compared with the control, CWA decreased IL-6, IL-8 and IL-22 levels and reduced neutrophil infiltration into the jejunum. CWA inhibited inflammation by down-regulating the TLR4-, MyD88- and NF-κB-dependent pathways. Additionally, CWA improved intestinal morphology by increasing villus and microvillus heights and enhancing intestinal barrier function by increasing tight junction (TJ) protein expression and augmenting wound-healing ability in intestinal epithelial cells. CWA also improved microbiota composition and increased short-chain fatty acid (SCFA) levels in feces. By contrast, Enro not only disrupted the intestinal barrier but also negatively affected microbiota composition and SCFA levels in the intestine. In conclusion, CWA effectively attenuated inflammation, enhanced intestinal barrier function, and improved microbiota composition in the intestines of weaned piglets. These results suggest that CWA could be an effective and safe therapy for diarrhea or other intestinal diseases in young mammals.

Diarrhea is a leading cause of death among children under 5 years of age, and causes death in more than one in ten children (approximately 800,000) per year, mainly in developing countries (WHO, 2015). Diarrhea typically occurs during the weaning period, which is a critical and stressful stage for young mammals^1^,^2^. Weaning causes reductions in body weight gain, decreases in nutrient absorption, disruptions to immune homeostasis and damage to barrier function^3^,^4^,^5^,^6^, resulting in susceptibility to pathogen infection and diarrhea^7^. Antibiotics have been effectively used to treat diarrhea in children and other animal neonates after weaning in past decades. Unfortunately, the widespread use of antibiotics has increased bacterial resistance, leading to delayed administration of effective therapy as well as morbidity and mortality in both humans and animals^8^,^9^. As reported, antimicrobial-resistant infections cause more than 700,000 deaths each year globally: at least 23,000 in the USA and 175,000 in the EU^10^,^11^. Furthermore, the development of new antibiotics has slowed down, and few antibiotics have been approved by the FDA in recent years^10^. Moreover, recent studies have reported that therapeutic or sub-therapeutic antibiotic treatments in early life have long-term consequences on intestinal microbiota composition and metabolic homeostasis in the host^12^,^13^. All this makes it clear that it is urgent to search effective and safe antimicrobial agents.

Antimicrobial peptides (AMPs) are short cationic molecules that serve as a host defense against microbial infection^14^,^15^. It is not easy for bacteria to develop resistance to AMPs because they work through a membrane-disrupting mechanism; therefore, they are considered a promising alternative to traditional antibiotics^16^. Cathelicidin peptides, such as human LL-37 and mouse CRAMP, exhibit antibacterial, antifungal and antiviral functions^17^. These peptides not only kill microbes directly but also modulate the immune system of the host^9^,^15^. LL-37 was shown to enhance the defenses of rats against pathogen infection, and CRAMP was found to ameliorate colonic colitis in dextran sulfate sodium (DSS)-induced mice^18^. Cathelicidin-WA (CWA), an AMP derived from the endemic genera *Bungarus fascia*, is considered a potent alternative to antibiotics^19^,^20^. Previous studies have shown that CWA had strong antimicrobial activities against ciprofloxacin-resistant pathogens and enteric pathogens isolated from the feces of piglets with diarrhea^21^,^22^. Our studies have revealed that CWA is highly unstable in gastrointestinal tract, but remains substantially intact in serum; the *in vivo* imaging results showed that intraperitoneal injection with CWA could be absorbed into the systemic circulation and availability to the intestine. Furthermore, CWA showed immunoregulatory capabilities *in vivo* and *in vitro*^17^.

The objective of this study was to investigate whether the CWA could be an effective therapy for diarrhea during weaning and to explore the immunoregulatory and epithelial barrier protective properties of CWA in the intestine of weaned piglets.

## Materials and Methods

### Peptide synthesis

CWA was synthesized by GL Biochem (Shanghai, China) as previously described^23^. Peptides were purified at greater than 95% purity via semi-preparative HPLC and characterized by analytical HPLC (Agilent 121 Technologies, CA, USA). The molecular weight of CWA was confirmed using a Thermo Finnigan LCQ ion trap mass spectrometer (Thermo Finnigan, CA, USA). CWA was prepared in saline before injection.

### Animals and sample collection

All experiments were approved by the Animal Care Committee of Zhejiang University and were conducted in accordance with the Guidelines for the Care and Use of Agricultural Animals for Research and Teaching at Zhejiang University. A total of 108 piglets with clinical diarrhea were selected from 1260 piglets (Duroc × Landrace × Yorkshire) at 3 d after weaning (25 ± 2 d). The piglets were randomly assigned to 1 of 3 treatments based on body weight, gender and diarrheal index. In total, there were 6 pens for each treatment, with 6 piglets per pen. The piglets were treated by intraperitoneal injection with normal saline (control), 0.6 mg/kg CWA (the dosage was based on results from LPS-induced mice and pre-experiment in diarrheal piglets), or 2.5 mg/kg enrofloxacin (Enro) once a day for 4 d. All piglets were provided with access to the diet (NRC1998) and water *ad libitum*. The diarrheal index was scored according to a fecal consistency scoring system (0, normal; 1, soft feces; 2, mild diarrhea; and 3, severe diarrhea) as described before^24^. A fecal score of 2 or 3 was considered clinical diarrhea. Fresh feces from each pen was collected and stored immediately at −20 °C. One piglet from each pen was randomly euthanized (n = 6 per treatment). Blood samples were collected from the anterior vena cava into coagulation accelerator tubes. Serum was obtained after centrifugation at 3000× g for 10 min at 4 °C. Samples of the middle duodenum, middle jejunum, distal ileum and middle colon were collected for analysis.

### Enzyme-linked immunosorbent assay (ELISA)

The levels of IL-6, IL-8 and IL-22 in serum and the jejunum were determined using ELISA kits (Raybiotech, GA, USA). In addition, the level of D-Lactate (D-Lac) in serum was detected using an ELISA kit (R&D, USA). Samples were measured according to the manufacturers’ instructions.

### Analysis of intestinal morphology

Intestinal tissues were fixed in 4% paraformaldehyde and embedded in paraffin. Sections of 5-μm thickness were stained with hematoxylin and eosin (H&E). Images were acquired using a DM3000 microscope (Leica, Wetzlar, Germany). Villous height and crypt depth were measured using Image-Pro software (MediaCybernetics, MD, USA) as previously described^25^. Histopathologic damage scores were determined under blinded conditions using a histologic injury scale (0, normal mucosal villi; 1, subepithelial Gruenha-gen’s space; 2, extension of subepithelial space with moderate lifting of epithelial layer from the lamina propria; 3, massive epithelial lifting down the sides of villi; 4, denuded villi with lamina propria and dilated capillaries exposed; and 5, digestion and disintegration of lamina propria) as previously described^23^.

### Scanning electron microscopy (SEM)

Jejunum tissue was fixed with 2.5% glutaraldehyde overnight and then with 1% OsO4 for 1 h. The jejunum specimens were then dehydrated in a graded series of ethanol (30%, 50%, 70%, 80%, 90%, 95% and 100%) for 20 min at each step and then transferred into a mixture of alcohol and iso-amyl acetate (v:v = 1:1) for 30 min and iso-amyl acetate for 1 h. The specimens were then dehydrated in a Hitachi Model HCP-2 critical point dryer with liquid CO_2_. The dehydrated specimens were coated with gold-palladium and visualized using a Philips Model SU8010 FASEM (HITACHI, Japan).

### Transmission electron microscopy (TEM)

Microvilli and epithelial cell junctions were observed by TEM. Jejunum tissue was fixed, dehydrated, and transferred into pure acetone for 20 min. The specimens were placed in a mixture of pure acetone and Spurr resin mixture (1:1 for 1 h and 1:3 for 3 h) and then transferred into Spurr resin mixture overnight. The tissue sections were placed in capsules containing embedding medium and heated at 70 °C for 9 h. The sections were then stained with uranyl acetate and alkaline lead citrate for 15 min and visualized via TEM (Model H-7650, HITACHI, Japan).

### Immunohistochemistry

Briefly, sections of jejunum were deparaffinized and rehydrated. The sections were submitted to antigen retrieval using EDTA buffer (pH = 9.0) and a microwave and then incubated in 3% hydrogen dioxide in the dark for 20 min. Following this, the sections were incubated with a primary antibody (1:200 dilution) against myeloperoxidase (MPO; Google Biotechnology Inc., Wuhan, China) to detect neutrophil infiltration. Sections were then incubated with a secondary antibody (1:200 dilution) and treated with DAB substrate. Nuclei were stained with Harris hematoxylin. Images were obtained using a DM3000 microscope.

### Real-time PCR

Total RNA was extracted using TRIzol reagent (Invitrogen, USA). RNA quantity and quality were determined using a NanoDrop 2000 spectrophotometer (Thermo Fisher Scientific, MA, USA). cDNA was obtained using 2 μg RNA. Real-time PCR was performed using a StepOne Plus^TM^ system (Applied Biosystems, CA, USA). The primers used for real-time PCR are listed in Table 1. Each reaction included 5 μL FastStart Universal SYBR Green Master Mix (Roche, Switzerland), 0.5 μL forward primer (10 μM), 0.5 μL reverse primer (10 μM) and 4 μL 10-fold diluted cDNA. The thermocycler protocol consisted of 10 min at 95 °C and 40 cycles of 10 s at 95 °C and 35 s at 60 °C, and melt curves were added. GAPDH and 18 S were used as housekeeping genes. mRNA relative expression was calculated using the 2^−ΔΔCt^ method.

### Western blotting

Total protein was extracted with lysis buffer (KeyGEN, Nanjing, China). Protein supernatant was separated by 10% SDS-PAGE and transferred onto a nitrocellulose membrane. After blocking with 5% skimmed milk powder, the membrane was incubated with the appropriate primary antibodies overnight at 4 °C, followed by incubation with a horseradish peroxidase (HRP)-conjugated secondary antibody for 1 h. Bands were detected using ECL (CliNX, Shanghai, China). Band intensity was quantified using ImageJ software. Primary antibodies for β-actin (Abcam, MA, USA), MyD88, phosphorylated NF-κB (Santa Cruz, CA, USA), phosphorylated IκB-α, phosphorylated AKT (Epitomics, USA), TLR4, zonula occluden-1 (ZO-1), occludin, and claudin-1 (Abcam) were used in this study.

### Microbial composition analysis

Genomic DNA was extracted from feces using a Fecal DNA Kit (SimGEN, Hangzhou, China). The DNA was quantified using a NanoDrop 2000 spectrophotometer. Quantitative measurements of total bacteria (forward: CGGTGAATACGTTCYCGG; reverse: GGWTACCTTGTTACGACTT), *Escherichia coli* (forward: CATGCCGCGTGTATGAAGAA; reverse: CGGGTAACGTCAATGA GCAAA), and *Lactobacillus* (forward: CGATGAGTGCTAGGTGTTGGA; reverse: CAAGATGTCAAGACCTGGTAAG) were performed by real-time PCR using a StepOne Plus^TM^ System as previously described^25^.

### Gas chromatographic analysis

Concentrations of short-chain fatty acids (SCFAs) in feces were determined via gas chromatography (GC-8 A, Shimadzu Corp., Kyoto, Japan). Briefly, 1 g of feces was mixed with 5 mL ddH_2_O and then centrifuged for 15 min at 10,000× g and 4 °C. Then, 1 mL of supernatant was mixed with 20 μL orthophosphoric acid (85%) for 1 h at 4 °C and centrifuged for 15 min at 12,000× g and 4 °C. The supernatant was transferred into a gas chromatography vial, and 2 μL of supernatant was injected into a 2-m × 3-mm glass column packed with Porapak Q (80 mesh; Agilent Technologies Inc., Santa Clara, CA, USA) as previously described^26^. SCFA concentrations were normalized to feces weight as μmol/g.

### Cell culture

Cells were cultured in RPMI-1640 or DMEM-F12 medium (Invitrogen) supplemented with 10% fetal bovine serum (Gibco) and antibiotics (100 U/mL penicillin and 100 μg/mL streptomycin sulfate) at 37 °C with 5% CO_2_ in a humidified incubator before treatment.

Porcine macrophage cells (3D4/2) were incubated for 6 h either with medium only or with 1 μg/mL lipopolysaccharide (LPS, *Escherichia coli* strain O111:B4, Sigma-Aldrich) after pretreatment with Enro (5, 10, 20, or 40 μg/mL) or CWA (5, 10, 20, or 40 μg/mL) for 12 h. Small-interfering RNA (siRNA) molecules targeting pig TLR4 or MyD88 were designed by Yingrun Biotechnology Co., Ltd. (Changsha, China). The 3D4/2 cells were transfected with 1 μg/mL siRNA using Lipofectamine-2000 (Invitrogen) for 6 h. Then, the transfected cells were incubated with medium only, LPS (1 μg/mL for 6 h), or CWA + LPS (pretreated with 20 μg/mL CWA for 12 h and then 1 μg/mL LPS for 6 h). Culture supernatant was collected for ELISA.

Porcine jejunal epithelial cells (IPEC-J2) were cultured in transwell dishes for at least 21 d until their transepithelial electrical resistance (TER) was stable. Monolayers of IPEC-J2 cells were incubated for 12 h with serum-free medium, LPS (1 μg/mL), or CWA + LPS (pretreated with 20 μg/mL CWA for 12 h and then 1 μg/mL LPS added) in the upper layer. TER was measured using a Millicell ERS-2 epithelial volt-ohm meter (Millipore MERS00002, USA). Cells were collected for Western blotting.

Caco-2 cells were cultured in 35-mm dishes. Caco-2 monolayers were scraped with 200-μL pipette tips as previously described^27^. The scraped Caco-2 cells were incubated with medium only or 20 μg/mL CWA. Images were obtained at 0 h, 48 h and 96 h. Wound width was measured at 48 h.

### Statistical analysis

Statistical analysis was performed using one-way ANOVA or Student’s t-test with SPSS 16.0 software (SPSS Inc., Chicago, IL, USA). Differences were considered significant at *P* < 0.05.

## Result

### CWA effectively attenuated diarrhea and systematic inflammation in weaned piglets

**Figure 1:**
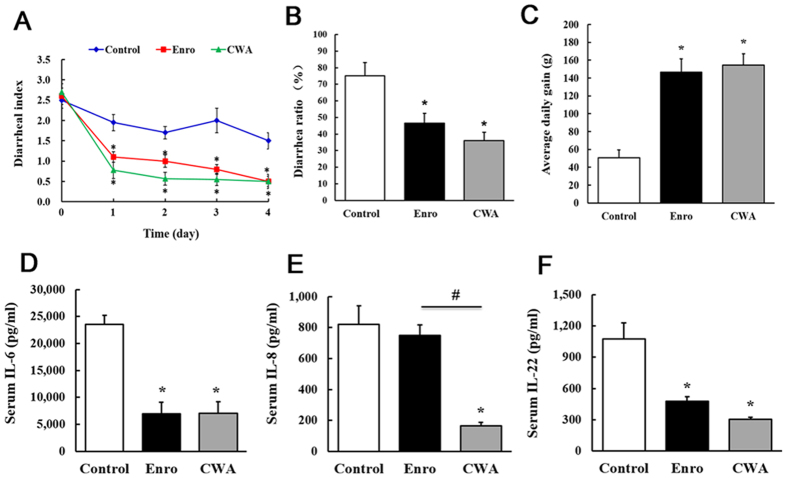
Cathelicidin-WA attenuated diarrhea and reduced serum pro-inflammatory cytokine levels in weaned piglets. (**A**) The diarrheal index was measured using fecal consistency scoring (0, normal; 1, soft feces; 2, mild diarrhea; and 3, severe diarrhea). (**B**) A fecal score of either 2 or 3 was considered to represent clinical diarrhea, and the diarrhea ratio was calculated. (**C**) Average daily gain. Serum levels of the pro-inflammatory cytokines IL-6 (**D**), IL-8 (**E**), and IL-22 (**F**) were determined by ELISA. All data are expressed as the mean ± SEM (n = 6). Differences were determined by one-way ANOVA. Enro, Enrofloxacin; CWA, Cathelicidin-WA. *P < 0.05 compared with control; ^#^*P* < 0.05 CWA compared with Enro.

Both CWA and Enro effectively decreased the diarrheal index and the diarrheal ratio and increased body weight gain compared with the control (Fig. 1A–C). Furthermore, CWA and Enro showed no significant differences in the diarrheal index, the diarrhea ratio, or body weight gain. Compared with the control, CWA treatment reduced levels of the pro-inflammatory cytokines IL-6, IL-8 and IL-22 in serum, whereas Enro treatment reduced levels of IL-6 and IL-22 (Fig. 1D–F). These data indicate that the CWA may be an effective alternative to antibiotics for the treatment of diarrhea in weaned piglets.

### CWA improved intestinal morphology and integrity in weaned piglets

**Figure 2:**
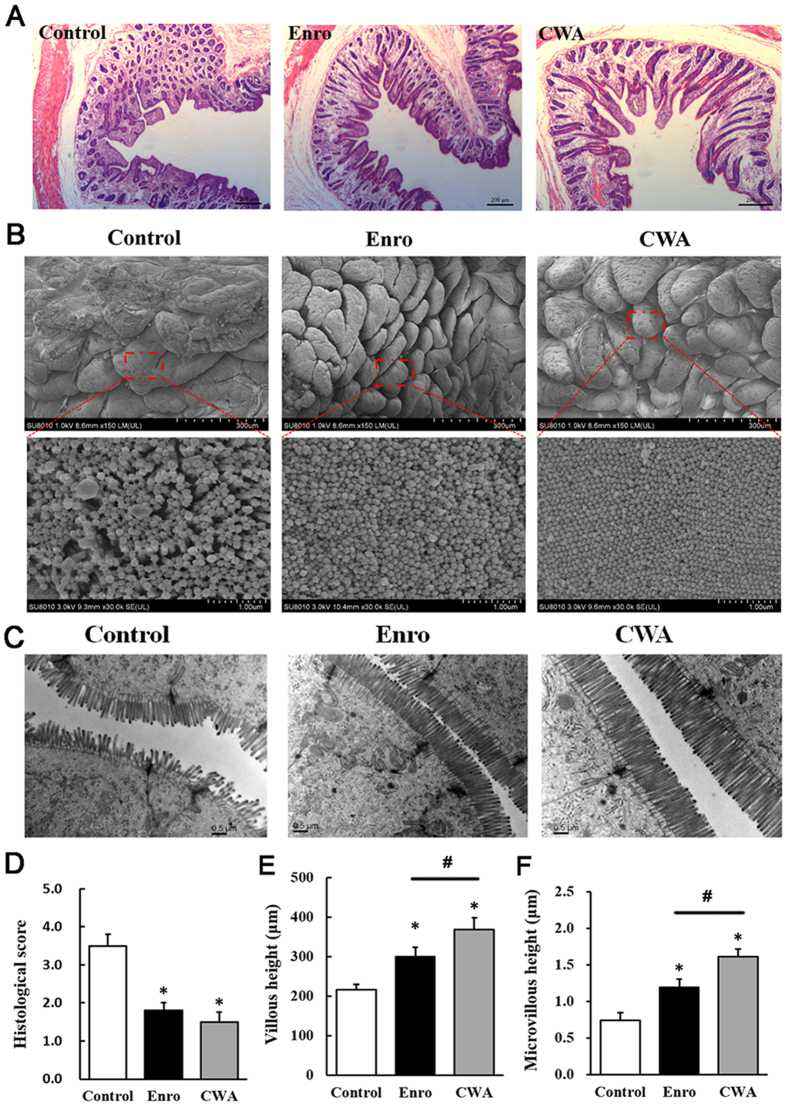
Cathelicidin-WA improved intestinal morphology. Jejunum sections were used to analyze intestinal morphology. (**A**) Stained with H&E (bars, 200 μm). (**B**) SEM images (upper, 150×; lower, 30,000×). (**C**) TEM images (bars, 0.5 μm). (**D**) Histological scores were determined as described in the *Materials and Methods*. (**E**) Villous height in the jejunum. (**F**) Microvillus height was measured using TEM. All data are expressed as the mean ± SEM (n = 6). Differences were determined by one-way ANOVA. Enro, Enrofloxacin; CWA, Cathelicidin-WA. *P < 0.05 compared with control; ^#^*P* < 0.05 CWA compared with Enro.

CWA treatment increased villous height in the jejunum compared with control and Enro treatments (Fig. 2A,E); this result was confirmed via SEM at 150× magnification (Fig. 2B). In addition, surface damage to villi in the jejunum was alleviated by CWA and Enro treatments (Fig. 2B, upper). Interestingly, both CWA and Enro increased the quantity of microvilli in the jejunum (Fig. 2B,C). Furthermore, CWA increased the height of microvilli in the jejunum compared with control and Enro (Fig. 2C,F). These results suggest that CWA effectively improved intestinal morphology and integrity in weaned piglets with clinical diarrhea.

### CWA suppressed intestinal inflammation via TLR4-, MyD88-, and NF-κB-dependent pathways

**Figure 3:**
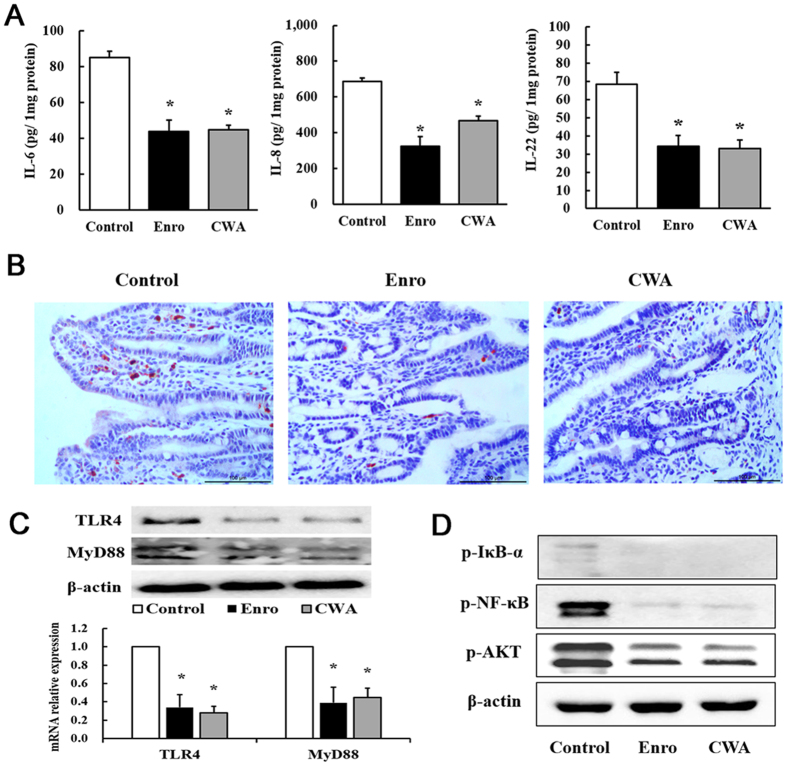
Cathelicidin-WA suppressed jejunal inflammation. (**A**) Levels of the pro-inflammatory cytokines IL-6, IL-8, and IL-22 in the jejunum. (**B**) Neutrophil infiltration into the mucosa of the jejunum was determined by immunohistochemical staining with an antibody against MPO (bars, 100 μm). (**C**) Relative mRNA expression and protein levels of TLR4 and MyD88 in the jejunum. (**D**) The phosphorylation of NF-κB, IκB-α, and AKT was determined by Western blotting. All data are expressed as the mean ± SEM (n = 6). Differences were determined by Student’s t-test. Enro, Enrofloxacin; CWA, Cathelicidin-WA. *P < 0.05 compared with control.

**Figure 4:**
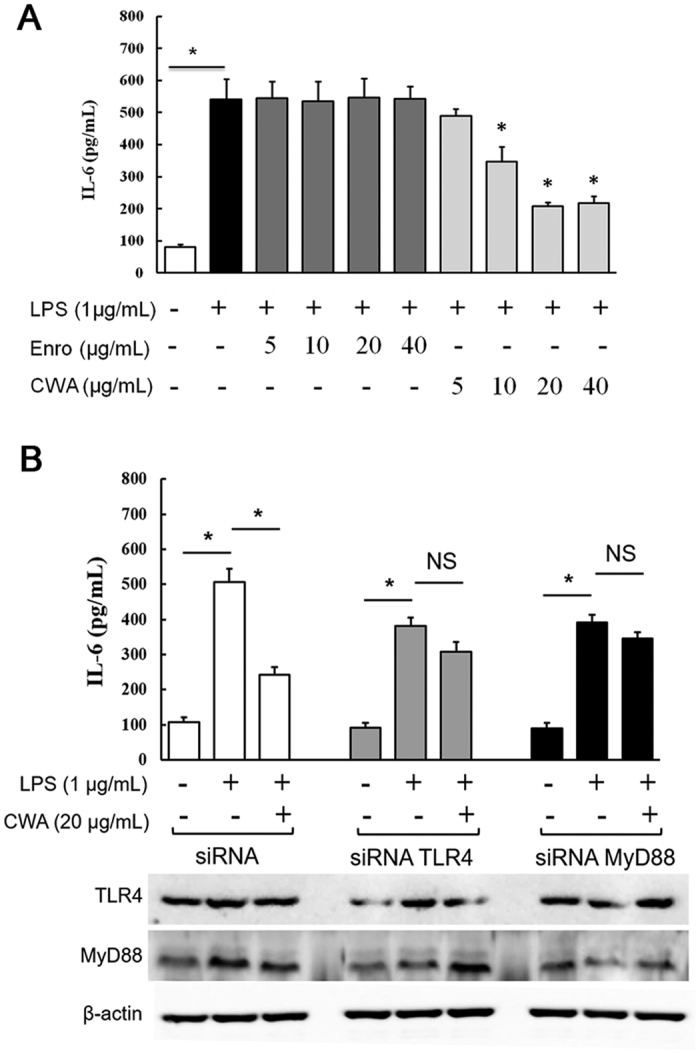
Cathelicidin-WA ameliorated LPS-induced inflammation via TLR4- and MyD88-dependent signaling pathways. (**A**) The effects of CWA and Enro on IL-6 expression in LPS-induced macrophages. (**B**) The effects of CWA on LPS-induced IL-6 expression in TLR4- and MyD88-silenced macrophages. All data are expressed as the mean ± SEM (n = 6). Differences were determined by one-way ANOVA. Enro, Enrofloxacin; CWA, Cathelicidin-WA. *P < 0.05 compared with LPS; NS, not significant, *P* > 0.05.

Levels of the pro-inflammatory cytokines IL-6, IL-8, and IL-22 in the jejunum were detected by ELISA. Both CWA and Enro significantly inhibited IL-6, IL-8 and IL-22 production in the jejunum compared with the control (Fig. 3A). In addition, immunohistochemistry results demonstrated that both CWA and Enro effectively decreased neutrophil infiltration in the mucosa of the jejunum compared with the control (Fig. 3B). Compared with the control, both CWA and Enro decreased the gene and protein expressions of TLR4 and MyD88 in the jejunum (Fig. 3C). Consequently, phosphorylation of NF-κB, IκB-α and AKT was suppressed in the jejunum following treatment with CWA and Enro (Fig. 3D). There were no significant differences between CWA and Enro regarding the suppression of intestinal inflammation *in vivo* in this study. However, *in vitro*, although Enro had no effects on LPS-induced expression of the pro-inflammatory cytokine IL-6 in porcine macrophages, CWA showed excellent inhibition of IL-6 production in a concentration-dependent manner (Fig. 4A). Furthermore, using siRNA-mediated knockdown, we found that CWA had no significant effects on LPS-induced IL-6 expression in MyD88-silenced or TLR4-silenced macrophages (Fig. 4B). These findings indicate that CWA shows excellent suppression of intestinal inflammation and may be involved in TLR4-, MyD88- and NF-κB-dependent pathways.

### CWA enhanced intestinal barrier functions

**Figure 5:**
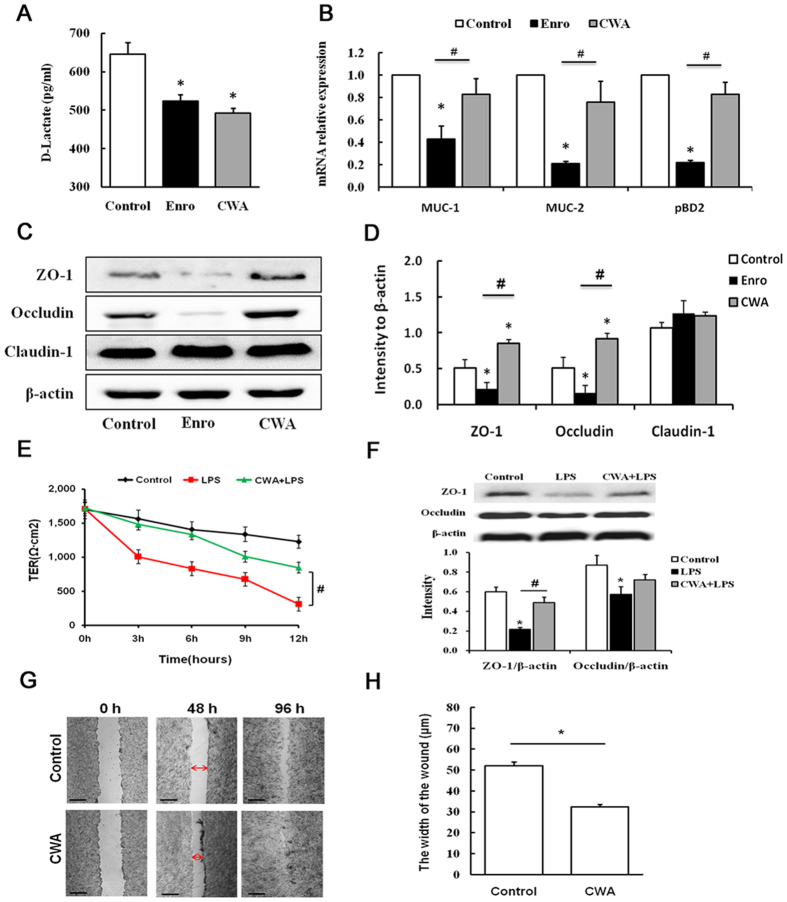
Cathelicidin-WA enhanced intestinal barrier function. (**A**) D-Lactate level in serum. (**B**) Relative mRNA expression levels of MUC-1, MUC-2 and pBD2 in the jejunum. (**C**,**D**) Protein expression levels of ZO-1, occludin and claudin-1 in the jejunum as determined by Western blotting. The effects of CWA on TER (**E**) and on the expression of ZO-1 and occludin proteins (**F**) in IPEC-J2 cell monolayers. (**G**,**H**) After being submitting to a scraping assay, Caco-2 cells were incubated with medium only or with 20 μg/mL CWA. Images were obtained at 0 h, 48 h and 96 h. Wound width was measured at 48 h. All data are expressed as the mean ± SEM (n = 6). Differences were determined by Student’s t-test. Enro, Enrofloxacin; CWA, Cathelicidin-WA. *P < 0.05 compared with the control; ^#^*P* < 0.05 CWA compared with Enro or LPS.

Both CWA and Enro decreased serum D-Lac levels compared with the control (Fig. 5A). Furthermore, Enro significantly reduced the expression of mucin-1 (MUC-1), mucin-2 (MUC-2) and porcine beta-defensin-2 (pBD2) mRNA in the jejunum compared with the control and CWA (Fig. 5B). In addition, we performed Western blot analysis to detect the expression of ZO-1, occludin and claudin-1. Although Enro had no significant effects on claudin-1 expression, ZO-1 and occludin expression levels in the jejunum were significantly reduced by Enro compared with the control (Fig. 5C,D). However, CWA significantly increased ZO-1 and occludin expression in the jejunum compared with the control (Fig. 5C,D). To verify these findings, we tested the effects of CWA on epithelial barriers *in vitro*. We found that CWA effectively ameliorated LPS-induced decreases in TER in IPEC-J2 cells (Fig. 5E). Furthermore, the expression levels of ZO-1 and occludin proteins in LPS-induced IPEC-J2 cells were increased by CWA (Fig. 5F). Interestingly, CWA significantly reduced would width at 48 h in a scrape assay in Caco-2 cells (Fig. 5G,H), suggesting that CWA could be beneficial for healing damage to intestinal epithelial cells. Collectively, CWA enhanced the intestinal barrier by increasing the expression of TJ proteins and augmenting wound healing ability. By contrast, Enro treatment may disrupt the intestinal barrier by decreasing the expression of TJ proteins and antimicrobial proteins.

### CWA improved microbial composition and SCFA levels in feces

**Figure 6:**
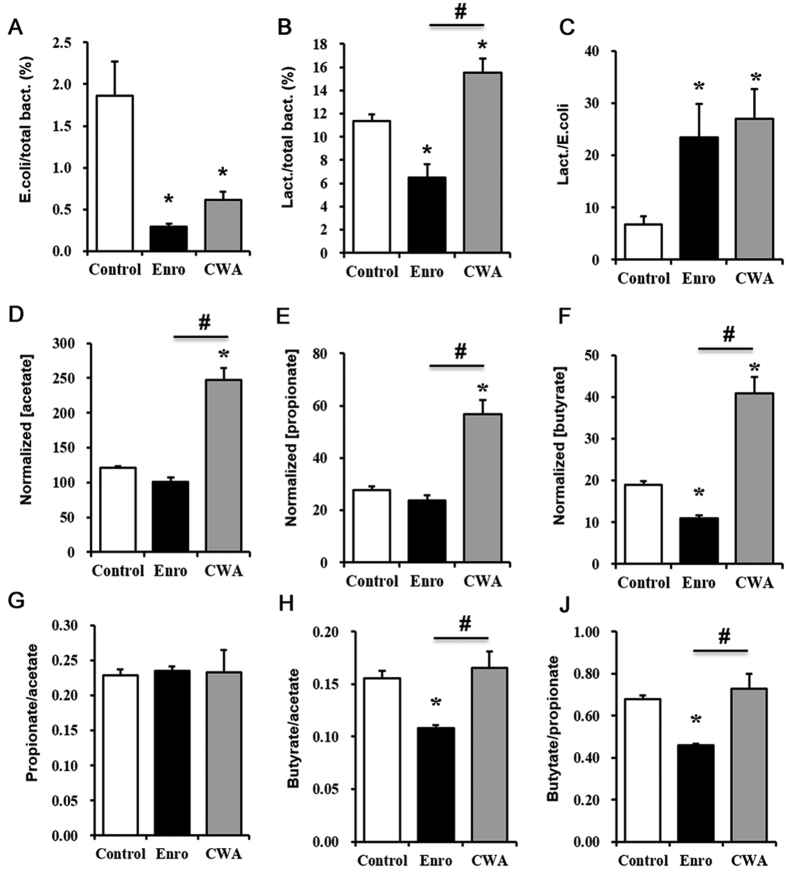
Cathelicidin-WA improved microbial composition and SCFA levels in feces. Ratios of *Escherichia coli* to total bacteria (**A**), *Lactobacillus* to total bacteria (**B**), and *Lactobacillus* to *Escherichia coli* (**C**) in feces as detected by qPCR. The concentrations of acetate (**D**), propionate (**E**), and butyrate (**F**) in feces were determined by GS, and the data were normalized to feces weight (per gram). Ratios of propionate/acetate (**G**), butyrate/propionate (**H**) and butyrate/acetate (**J**). All data are expressed as the mean ± SEM (n = 6). Differences were determined by one-way ANOVA. Enro, Enrofloxacin; CWA, Cathelicidin-WA. *P < 0.05 compared with the control; ^#^*P* < 0.05 CWA compared with Enro.

To evaluate CWA’s effects on intestinal epithelial barrier-microbiota interactions, we evaluated microbiota composition and SCFAs in the feces of weaned piglets following CWA treatment. Both CWA and Enro decreased the ratio of *Escherichia coli* to total bacteria and increased the ratio of *Lactobacillus* to *Escherichia coli* in feces compared with the control (Fig. 6A,C). The ratio of *Lactobacillus* to total bacteria in feces was deceased by Enro but increased by CWA compared with the control (Fig. 6B). Furthermore, CWA increased acetate, propionate and butyrate concentrations, but it did not alter the ratios of propionate/acetate, butyrate/acetate and butyrate/propionate in feces compared with the control (Fig. 6D–J). By contrast, Enro not only decreased butyrate concentration but also reduced the ratios of butyrate/propionate and butyrate/acetate in feces compared with the control and CWA (Fig. 6F,H,J).

## Discussion

In this study, we demonstrated that the CWA can effectively attenuate postweaning diarrhea by suppressing inflammation, enhancing barrier function, and improving microbiota composition and SCFA levels in the intestine of piglets. By contrast, the antibiotic Enro disrupted barrier function, changed microbiota composition and negatively impacted SCFA levels in the intestine. Collectively, these data indicate that CWA could be an effective and safe therapy for diarrhea or other intestinal diseases and may have the potential to reduce antibiotic use in young mammals.

Diarrhea is a leading cause of death in young children, especially during the weaning period. Antibiotics are usually used for the treatment of diarrhea. In this study, we selected weaned piglets with clinical diarrhea as models to compare the effects of CWA and the antibiotic Enro on diarrhea in young mammals. To reduce the influence of bacterial resistance, we chose to evaluate Enro in this study, which is currently approved by the FDA and has strong antibacterial activity against both Gram-negative and Gram-positive bacteria. Surprisingly, we found that CWA attenuated diarrhea in weaned piglets as effectively as Enro. This is the first study to demonstrate that AMPs and antibiotics can produce similar therapeutic efficacy against postweaning diarrhea or other intestinal diseases in young mammals.

Although there are varied causes for diarrhea, intestinal inflammation is usually always present. Pro-inflammatory cytokine levels, such as those of IL-6 and TNF-α, are typically increased in the small intestines of piglets after weaning^27^. In this study, we demonstrated that CWA suppressed intestinal inflammation by down-regulating the TLR4-, MyD88- and NF-κB-dependent pathways in weaned piglets with diarrhea. Consistent with our results, previous studies have shown that cathelicidin peptides can decrease pro-inflammatory cytokine levels by inhibiting the NF-κB signaling pathway in LPS-induced macrophages and in mice^23^,^28^. The anti-inflammatory effects of the human-derived cathelicidin peptide LL-37 have been widely investigated^29^,^30^,^31^. LL-37 regulates inflammatory responses to pathogens, LPS or other TLR agonists by inhibiting NF-κB^32^. However, cathelicidins appear to possess both anti-inflammatory and pro-inflammatory properties in immunity^33^. For instance, LL-37 has been shown to promote the expression of the chemokine IL-8 34. In addition, cathelicidins are directly chemotactic for various innate immune cells, such as dendritic cells and macrophages^17^. Cathelicidins such as mouse CRAMP and human LL-37 play important roles in autoimmune diseases. CRAMP is protective in non-obese diabetes and can convert inflammatory cells into regulatory immune cells via the EGFR and TGF-β signaling pathways^35^. These data indicate that CWA may also have multiple ways of modulating intestinal inflammation.

As Enro treatment also effectively suppressed intestinal inflammation in weaned piglets, the question arises of whether the anti-inflammatory capacity of CWA is only dependent on its antibacterial activity (such as with antibiotics). Thus, we evaluated the immunomodulatory properties of CWA and Enro in LPS-stimulated porcine macrophages *in vitro*. Interestingly, Enro had no effects on the LPS-induced expression of the pro-inflammatory cytokine IL-6, whereas CWA effectively suppressed IL-6 expression. The differential regulation of IL-6 expression by Enro and CWA in macrophages may be related to the antibiotic-independent immunomodulatory effects of cathelicidins. Cathelicidins have been reported to suppress LPS-induced inflammation in macrophages via several mechanisms: binding to LPS; perturbation of the MyD88 signaling pathway; and inhibition of NF-κB translocation^35^,^36^. In this study, CWA was used to treat macrophages for 12 h and then removed before LPS treatment; thus, any anti-inflammatory activities of CWA were independent of LPS binding activity in macrophages. In addition to inactivating LPS through binding, a lactoferrin peptide was also shown to modulate LPS-induced inflammatory responses by directly affecting the MyD88-NF-κB signaling pathway in macrophages^37^. Consistent with these results, we found that CWA-mediated suppression of IL-6 expression was a direct result of CWA’s effects on the TLR4 and MyD88 signaling pathways. Collectively, these data suggest that the mechanisms by which CWA inhibits inflammation could be mediated by the down-regulation of the TLR4-, MyD88- and NF-κB-dependent pathways independently of CWA’s antibacterial activity. This effect may explain why CWA (0.6 mg/kg) and Enro (2.5 mg/kg) exhibited similar effects on the suppression of intestinal inflammation in weaned piglets with diarrhea.

Intestinal barrier damage is present in many intestinal diseases, such as diarrhea and inflammatory bowel disease (IBD). The epithelial barrier of the intestine is responsible for restricting the invasion of harmful substances, including microbes, toxins and LPS^38^. TJs are the most apical intercellular structures in the mucosa of the intestine and create semipermeable barriers that can separate different substances^39^. ZO-1, occludin and the claudins are the most important components of TJs in intestinal epithelial cells. In this study, we demonstrated that CWA enhanced epithelial barrier function by increasing the expression of ZO-1 and occludin in the intestine of weaned piglets with diarrhea. Consistently, previous studies have shown that AMPs such as pBD2 increase ZO-1, occludin and claudin-1 mRNA expression in the colon of DSS-induced mice^17^,^39^. We confirmed this result *in vitro* by showing that CWA ameliorated decreases in TER and improved the expression of TJ proteins in LPS-induced IPEC-J2 cells. By contrast, Enro treatment reduced the expression of TJ proteins and also inhibited the expression of mucins and pBD2 in the jejunum, suggesting that epithelial barrier function in the intestine is weakened by treatment with antibiotics. Decreased expression of TJ proteins could increase the permeability of the epithelial barrier, exacerbating inflammation^40^. The mucins and antimicrobial proteins that are secreted by intestinal epithelial cells provide defenses against pathogen invasion into the underlying lamina propria^13^,^41^. A previous study demonstrated that administration of the antibiotics neomycin, metronidazole and vancomycin significantly decreased the expression levels of antimicrobial proteins in intestinal epithelial cells^42^. A recent study also demonstrated that the antibiotic metronidazole compromised goblet cell function and inner mucus layer production and exacerbated *Citrobacter rodentium*-induced colitis in mice^13^. These data indicate that CWA treatment could enhance the intestinal barrier, whereas treatment with antibiotics might disrupt the intestinal barrier in young mammals.

Studies using knockout mice and germ-free mice have showed that intestinal mucins and antimicrobial proteins are major mediators of intestinal epithelial cell-commensal interactions, and their functions are largely affected by microbiota^41^,^43^. The different effects produced by CWA and Enro on the intestinal barrier may be related to changes in microbiota composition. As reported, dietary supplementation with AMPs can improve microbiota composition in the intestines of weaned pigs^44^,^45^,^46^. However, antibiotic treatments perturb this microbial composition and alter a host’s susceptibility to enteric infection^13^,^47^. In the current study, we found that CWA increased the ratio of *Lactobacillus* to total bacteria and increased SCFA levels in feces, whereas Enro decreased the ratio of *Lactobacillus* to total bacteria, the concentration of butyrate, and the ratios of butyrate/propionate and butyrate/acetate. These results provide evidence that antibiotics not only perturb microbial composition but also affect the metabolic capabilities of the microbiome in the intestines of a host^12^.

Given its effects on inflammation, the intestinal barrier and microbiota composition, CWA could be an effective therapy for many diseases linked to imbalances in homeostasis in the intestine, including obesity, IBD and colorectal cancer. The optimal dosage of CWA treatment for different type of diseases needs to be further evaluated. Additionally, the mechanisms underlying how CWA affects the expression of TJ proteins and enhances bacterial resistance need to be determined in the future.

In conclusion, CWA effectively attenuated intestinal inflammation, enhanced intestinal barrier function, and improved microbial composition and metabolic capabilities in the intestines of weaned piglets. Furthermore, our results demonstrated that antibiotic treatment perturbed epithelial barrier function and negatively altered microbiota composition in the intestine. This study indicates that CWA treatment could be an effective therapy for postweaning diarrhea in young mammals as well as a therapeutic strategy for other intestinal diseases, such as IBD and colorectal cancer.

## Additional Information

**How to cite this article**: Yi, H. *et al*. High therapeutic efficacy of Cathelicidin-WA against postweaning diarrhea via inhibiting inflammation and enhancing epithelial barrier in the intestine. *Sci. Rep.* **6**, 25679; doi: 10.1038/srep25679 (2016).

## Figures and Tables

**Table 1:** Primers for real-time PCR in this study.

Gene	Sequence (5′−3′)	Size(bp)	Accession number
TLR4	Forward: GCCATCGCTGCTAACATCATCReverse: CTCATACTCAAAGATACACCATCGG	108	NM_001113039.1
MyD88	Forward: TGGTAGTGGTTGTCTCTGATGAReverse: TGGAGAGAGGCTGAGTGCAA	80	NM_001099923.1
Mucin-1	Forward: ACACCCATGGGCGCTATGTReverse: GCCTGCAGAAACCTGCTCAT	68	NM_001204296.1
Mucin-2	Forward: CTGCTCCGGGTCCTGTGGGAReverse: CCCGCTGGCTGGTGCGATAC	100	XM_007465997.1
pBD2	Forward: CCAGAGGTCCGACCACTACAReverse: GGTCCCTTCAATCCTGTTGAA	88	AY506573.1
18 S	Forward: CCCACGGAATCGAGAAAGAGReverse: TTGACGGAAGGGCACCA	122	AY265350.1
GAPDH	Forward: ACTCACTCTTCCACTTTTGATGCTReverse: TGTTGCTGTAGCCAAATTCA	100	NM_001206359
